# Unveiling the factors shaping the distribution of widely distributed alpine vertebrates, using multi-scale ecological niche modelling of the bat *Plecotus macrobullaris*

**DOI:** 10.1186/s12983-014-0077-6

**Published:** 2014-10-29

**Authors:** Antton Alberdi, Ostaizka Aizpurua, Joxerra Aihartza, Inazio Garin

**Affiliations:** Department of Zoology and Animal Cell Biology, Faculty of Science and Technology, University of The Basque Country UPV/EHU, Sarriena auzoa s/n, 48940 Leioa, The Basque Country

**Keywords:** Alpine distribution, Alpine long-eared bat, Biogeography, Chiroptera, Distribution, Modelling, Mountain long-eared bat, Zoogeography

## Abstract

Several alpine vertebrates share a distribution pattern that extends across the South-western Palearctic but is limited to the main mountain massifs. Although they are usually regarded as cold-adapted species, the range of many alpine vertebrates also includes relatively warm areas, suggesting that factors beyond climatic conditions may be driving their distribution. In this work we first recognize the species belonging to the mentioned biogeographic group and, based on the environmental niche analysis of *Plecotus macrobullaris*, we identify and characterize the environmental factors constraining their ranges. Distribution overlap analysis of 504 European vertebrates was done using the Sorensen Similarity Index, and we identified four birds and one mammal that share the distribution with *P. macrobullaris.* We generated 135 environmental niche models including different variable combinations and regularization values for *P. macrobullaris* at two different scales and resolutions. After selecting the best models, we observed that topographic variables outperformed climatic predictors, and the abruptness of the landscape showed better predictive ability than elevation. The best explanatory climatic variable was mean summer temperature, which showed that *P. macrobullaris* is able to cope with mean temperature ranges spanning up to 16°C. The models showed that the distribution of *P. macrobullaris* is mainly shaped by topographic factors that provide rock-abundant and open-space habitats rather than climatic determinants, and that the species is not a cold-adapted, but rather a cold-tolerant eurithermic organism. *P. macrobullaris* shares its distribution pattern as well as several ecological features with five other alpine vertebrates, suggesting that the conclusions obtained from this study might be extensible to them. We concluded that rock-dwelling and open-space foraging vertebrates with broad temperature tolerance are the best candidates to show wide alpine distribution in the Western Palearctic.

**Electronic supplementary material:**

The online version of this article (doi:10.1186/s12983-014-0077-6) contains supplementary material, which is available to authorized users.

## Introduction

Studying the causes shaping distribution patterns is one of the major tasks in biogeography [1]. Many organisms exhibit similar distributions that despite their current ranges could be the result of different historical, ecological and physiological processes. Identifying the common factors constraining their range is key to understanding these geographic analogies. The joint characteristic attribute of alpine species is the geographic restriction to high mountain environments, which were covered with ice-sheets during long periods in the Pleistocene [2]. The study of the biogeographic patterns of Eurasian alpine species has mainly targeted plants and invertebrates [3-6] that due to either low or no mobility [7,8] are largely conditioned by edaphic and climatic factors [9]. Most vertebrates, however, are unique in their higher mobility, and due to their distinct ecological and evolutionary traits, the biogeographic patterns of vertebrates are likely to differ from those of plants and invertebrates.

The distribution patterns of high mountain vertebrates in the Western Palearctic vary considerably. Endemics restricted to one or few mountain systems are common mainly among vertebrates with low mobility [10,11]. Most volant species however show wider distributions. Many of them are ubiquitous and can be commonly found in other habitats in addition to alpine environments [12], and few arctic-alpine species show broad boreal distributions with isolated relict populations in alpine areas at lower latitudes [13]. Finally, there is a group of species sharing a broad geographic distribution restricted to Southern Palearctic mountain systems, hereafter referred to as palealpine species, whose distribution pattern has so far received little attention. The distribution of a single bat species, the alpine long-eared bat (*Plecotus macrobullaris*) is also known to fit this biogeographic pattern [14]. *P. macrobullaris* is a recently described species [15-17] known to forage in alpine meadows [18] and roost mainly in rock crevices and talus slopes [19]. Unlike other bat species in the Western Palearctic [20], this species has breeding populations in the subalpine and alpine belts of mountain systems in the South-western Palearctic [14].

The factors explaining why palealpine vertebrates like *P. macrobullaris* are restricted to mountain environments, yet absent from the rest of the region, have so far not been addressed. Although species inhabiting alpine environments are commonly regarded as cold-adapted species [21], this relationship is not always accurate. Whilst species with arctic-alpine distributions are physiologically restricted to cold conditions and remain active in the alpine belt during the winter [13], this is not necessarily the case for all alpine species [22]. In fact, the current distribution of many palealpine species also includes relatively warm areas in South-eastern Europe and the Middle East [23], suggesting that factors beyond climatic conditions likely dictate the distribution of these species.

Identifying the factors constraining geographic ranges requires using the correct resolution and extent [24] because the distribution of species is shaped by processes acting at different scales [25-27]. Climatic factors mainly drive the continental scale distribution due to the physiological limits of species [28]. Ecological features, instead, usually act more locally and require finer resolution [29,30]. Additionally, the effect of topographic factors such as slope or aspect also depends on scale [31]. Hence, a multiple resolution and extent approach allows the input of variables in the correct scale in which they act, providing more representative outputs of the environmental factors limiting the distribution of species [32].

In this study we test whether climatic variables or habitat and topographic factors are the main drivers of the distribution of palealpine vertebrates, so that the main factors constraining their distribution are identified. First, we identify the vertebrate species that exhibit palealpine distribution analysing range similarities. And secondly, in order to understand why palealpine species are closely linked to mountain environments, we apply our modelling approach at two spatial scales using *P. macrobullaris* as model species. At the broad-scale, across the Western Palearctic, we use coarse-grain presence records of the species for continental-level modelling, while at the fine-scale, around the Pyrenees, we use precise roosting records and finer spatial resolution to understand the factors shaping the local distribution.

## Results

**Figure 1:**
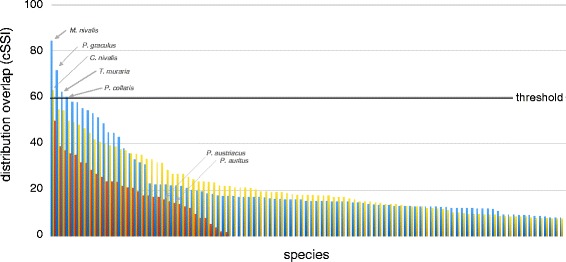
**Geographic overlap between the distribution of** ***P. macrobullaris*** **and the distributions of analysed European vertebrates.** Blue bars indicate birds, yellow bars indicate non-chiropteran mammals and red bars indicate bats. The names of the five species with the highest distribution resemblance, and the positions of *P. auritus* and *P. austriacus* are shown. Note that the species with cSSI values equal to 0 (distributions not-overlapped) are not shown and the list of species of birds and non-chiropteran mammals is limited to 100 species.

The Sørensen Similarity Index (SSI) value between the distribution area we generated based on updated distribution information of *P. macrobullaris* [14] and the area available at IUCN Red List was 0.66. Thus, that was the error-factor we used for calculating the corrected SSI (cSSI) values of the other species, since we consider it more adjusted to the actual distribution similarity between species (Figure 1). The distribution overlap analysis showed that four birds and a single mammal share the geographic distribution with *P. macrobullaris*, namely the birds *Montifringilla nivalis*, *Pyrrhocorax graculus*, *Tichodroma muraria*, *Prunella collaris* and the vole *Chionomys nivalis* (Figure 1). All these species showed cSSI values above 0.6, while the average cSSI values for birds and mammals were 0.15±0.15 and 0.12±0.14 respectively. The average cSSI value for bats was 0.18±0.12.

### Broad-scale distribution modelling

Analysed evaluators showed that the model with the best predictive ability was the one including one topographic (ABR), five climatic (B4, B10, B12, B15 and B17) and one habitat-related (LAND) variables, generated with a regularization multiplayer of β=1 (Table 1, Additional file 1: Table S1). The predictive value of abruptness out-performed elevation in all the analysed cases. The Spearman correlation values (ρ) between elevation and mean summer temperature were −0.97 in the regional scale and −0.43 in the continental scale.

**Table 1:** **Composition, evaluation scores (AUCtest, AICc and MPA) and variable contribution ranks of the best models**

**Broad-scale modelling**						
**Type**	**Model**	**β**	**AUC**_**test**_	**AICc**	**MPA**	**Variable contribution rank**
Climatic	B8, B10, B12, B17	1	0.881	2688.1285	0.3383	B10 (45.3) > B12 > B8 > B17
	B4, B10, B12, B15, B17	1	0.880	2619.7250	0.2972	B10 (43.7) > B12 > B17 > B15 > B4
Topographic	ABR, ELEV	1	0.873	2434.7099	0.365	ABR (84.4) > ELEV
	ABR, ELEV	2	0.866	2478.7364	0.343	ABR (86.1) > ELEV
Climatic + Topographic	B4, B8, B10, B12, B15, B17, ELEV, ABR	1	0.905	2542.8793	0.201	ABR (71.7) > ELEV > B10 > B17 > B12 > B15 > B4 > B8
	B4, B8, B10, B12, B15, B17, ABR	2	0.911	2409.3266	0.237	ABR (79.9) > B10 > B12 > B15 > B17 > B4 > B8
Climatic + Habitat	B4, B8, B10, B12, B15, B17, LAND	1	0.899	2426.4305	0.271	B10 (32.7) > B12 > LAND > B8 > B17 > B15 > B4
	B4, B10, B12, B15, B17, LAND	1	0.889	2472.1983	0.237	B10 (37.0) > B12 > LAND > B17 > B15 > B4
Topographic + Habitat	ELEV, ABR, LAND	1	0.873	2333.0673	0.321	ABR (86.7) > ELEV > LAND
	ABR, LAND	2	0.876	2379.0112	0.321	ABR (96.6) > LAND
Climatic + Topographic + Habitat	B4, B8, B10, B12, B15, B17, ABR, LAND	1	0.903	2334.3659	0.178	ABR (70.6) > B10 > B17 > LAND > B15 > B12 > B8 > B4
	**B4, B10, B12, B15, B17, ABR, LAND**	**1**	**0.912**	**2330.3513**	**0.208**	**ABR (75.1) > B10 > BIO12 > B17 > B15 > LAND > B4**
**Fine-scale modelling**						
**Type**	**Model**	**β**	**AUC**_**test**_	**AICc**	**MPA**	**Variable contribution rank**
Topographic	ELEV, SLOP, ORI	1	0.9294	1087.2041	0.1381	ELEV (56.3) > SLO > ORI
	ELEV, SLOP	1	0.9298	1108.6065	0.1574	ELEV (54.9) > SLO
Habitat	DIS-URBAN, DIS-FOREST, DIS-ROCK	1	0.8968	1161.1387	0.2449	dis-rock (74.3) > dis-urban > dis-forest
	DIS-URBAN, DIS-ROCK	1	0.8943	1321.0146	0.2471	dis-rock (89.3) > dis-urban
Topographic + Habitat	ELEV, SLOP, ORI, DIS-URBAN, DIS-FOREST, DIS-ROCK	1	0.9429	1289.9253	0.0871	dis-rock (32.0) > SLO > ELEV > dis-urban > dis-forest > ORI
	**ELEV, SLOP, DIS-URBAN, DIS-ROCK**	**1**	**0.9560**	**1199.8611**	**0.1026**	**dis-rock (34.4) > SLO > ELEV > dis-urban**

Only the two models that obtained the best values in each variable-type combination are shown. The rest of the models can be found in Tables S1 and S2 in the Additional file 1. The contribution percentage of the best-ranked variable in each model is indicated in brackets. The selected models are marked in bold.

**Figure 2:**
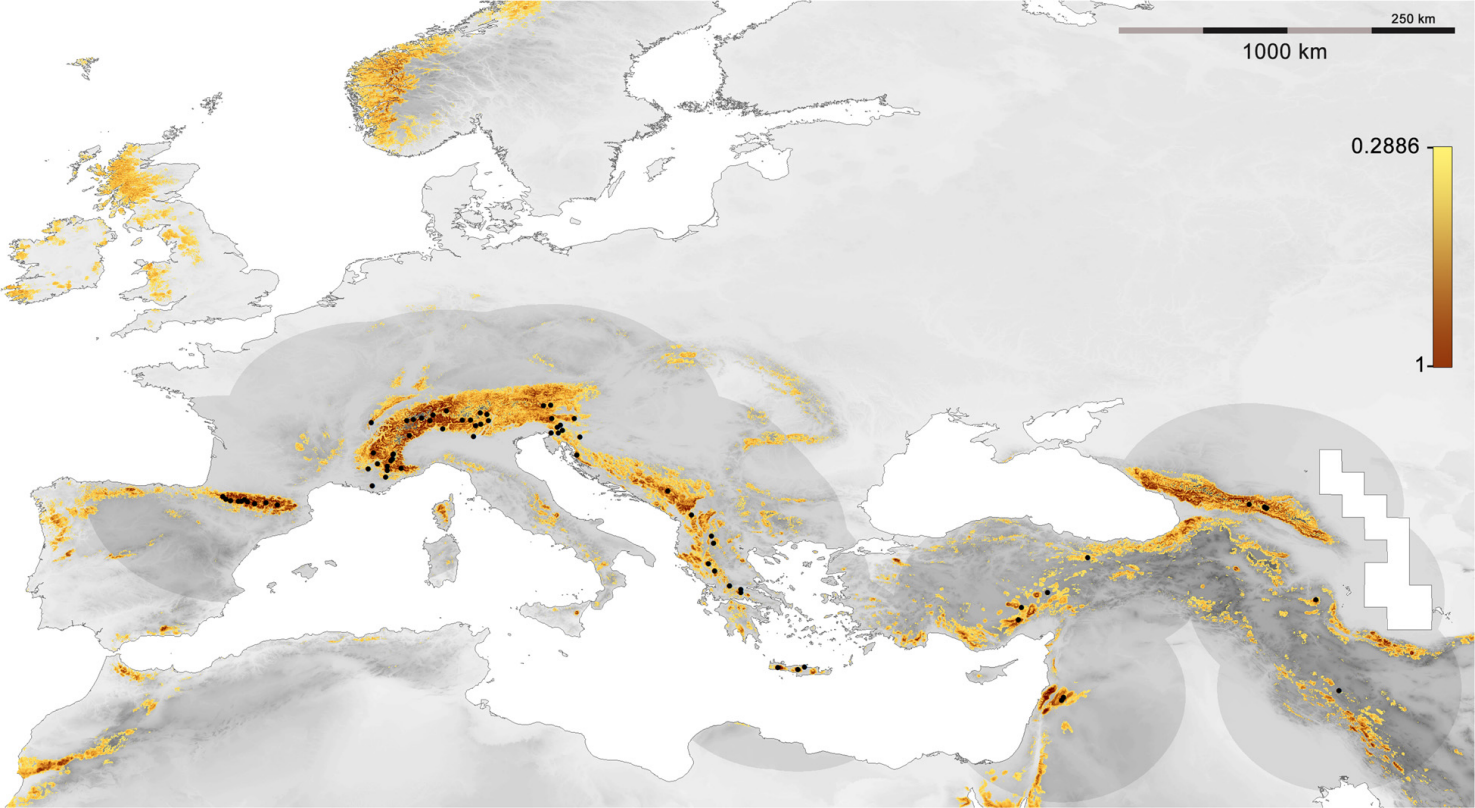
**Distribution model for** ***P. macrobullaris,*** **presented on a greyscale elevation map.** Only suitable areas (SV >0.2886) are shown in a colour gradient from light yellow (low suitability) to brown (high suitability). Presence location records used for modelling are represented by black dots and the darkened region is the area used for calibrating the model.

**Figure 3:**
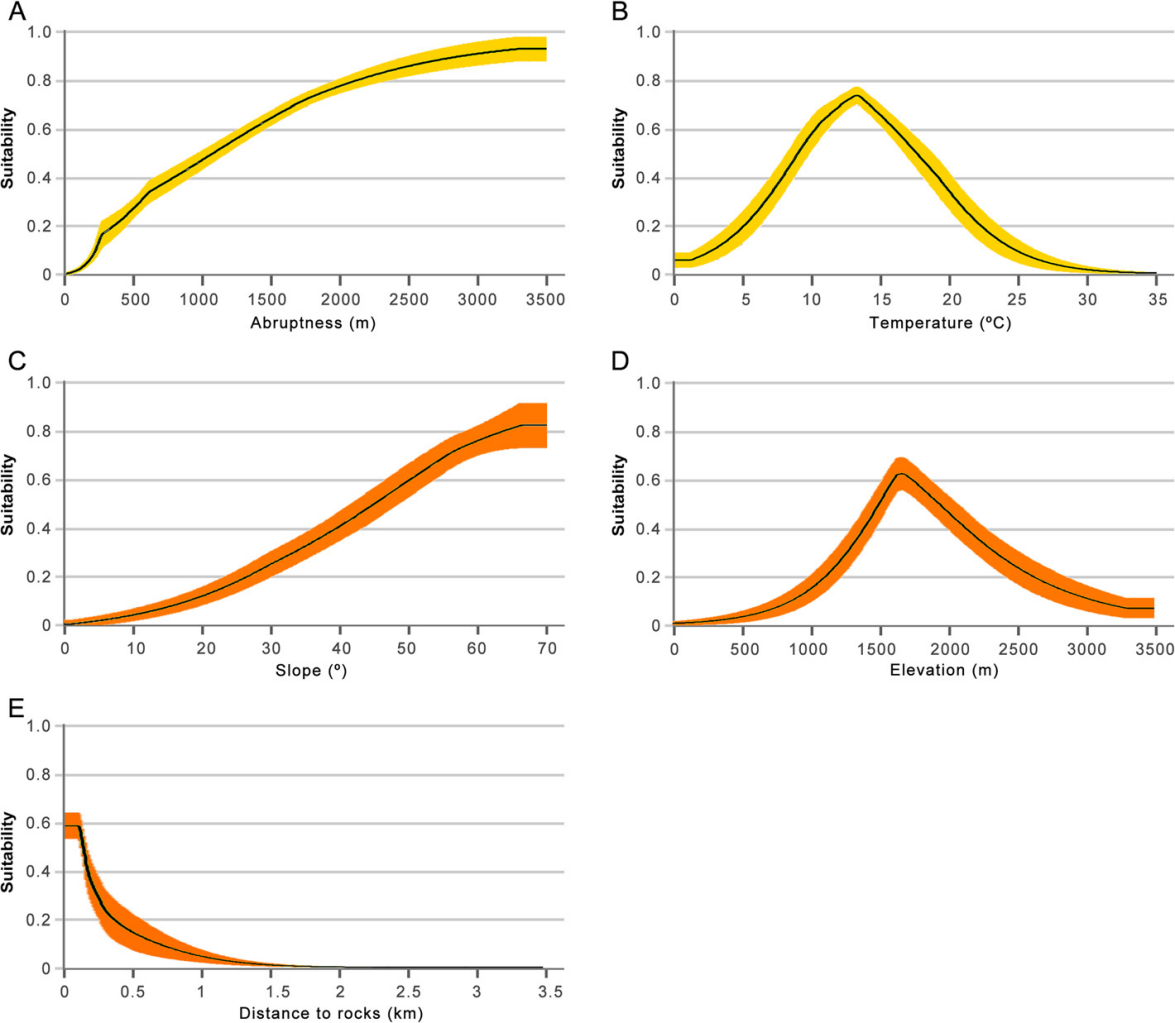
**Response curves of variables with the highest predictive ability. (A)** Abruptness and **(B)** mean temperature of the warmest quarter, as estimated by the broad-scale modelling; **(C)** slope, **(D)** elevation and **(E)** distance to rocks (DIS-ROCK), as estimated by the fine-scale modelling.

Areas with suitability values below 0.2886 (max SSS) were considered unsuitable, and 89.4% of the total known distribution records were located in suitable cells. The best model showed suitable areas in the main mountain ranges of the Southern Palearctic, as well as some areas of Northern Europe (Figure 2). All metrics indicated that abruptness was the variable with the best explanatory power (Table 2, Additional file 2: Figure S2). The second most important variable, based on all analysed metrics, was the mean temperature of the warmest quarter (B10), and the remaining climatic and habitat-related variables obtained lower values in all the evaluated metrics. The response curve of the best explanatory variable showed that suitability increased with abruptness, starting with zero probability of presence in flat regions (Figure 3A). The most suitable mean temperature of the warmest quarter was 14°C, but suitable values ranged from 6 to 22°C (Figure 3B).

**Table 2:** **Different metrics on the contribution of variables to the best models**

**Variable**	**Relative contribution**	**Permutation importance**	**Jackknife training gain with only variable**	**Jackknife training gain without this variable**
Broad-scale modeling				
ABR	75.10	45.45	1.05	1.05
B10	9.01	28.17	0.64	1.25
B12	3.86	7.34	0.59	1.34
B17	4.63	6.45	0.41	1.34
B15	2.34	2.28	0.09	1.34
LAND	3.70	1.57	0.20	1.33
B4	1.33	8.71	0.11	1.34
Fine-scale modeling				
DIS-ROCK	34.36	42.86	0.97	1.71
SLOPE	31.68	14.36	1.16	1.53
ELEV	25.50	28.49	1.02	1.68
DIS-URBAN	8.44	14.27	0.30	1.73

All values are averages of the 50 replicates of the best models. The relative contribution is obtained from the increase of the regularized gain when each variable is added to the model. The permutation importance is obtained by randomly permuting the values of that variable among the training points and measuring the decrease in training AUC produced by the permutation. The Jackknife training gain with only variable is the training gain that the model achieves when using only that variable, and the Jackknife training gain without this variable is the training gain that the model achieves when using the rest of variables except that one. Consequently, in the first three metrics larger values indicate higher contribution of variables to the model, while in the last one, the lower values indicate greater importance of variables to the model.

### Fine-scale roosting habitat suitability modelling

The best model was built using two habitat (distance to rocky and urban areas) and two topographic variables (elevation and slope) (Table 1, Additional file 1: Table S2). The ENM showed good predictive ability (AUC =0.956). The three main factors shaping roosting habitat suitability for *P. macrobullaris* were the distance to rock areas (DIS-ROCK), slope and elevation. DIS-ROCK was the variable with the highest relative contribution to the best model, and Jackknife tests stressed the importance of slope and elevation (Table 2, Additional file 2: Figure S3). The closer the rocks and higher the slope the greater the probability of finding suitable roosting sites (Figure 3C and E). The response curve for elevation showed suitable areas for roosting in an elevation range from 1300 to 2400 m, with maximum values between 1500 and 2000 m (Figure 3D).

## Discussion

**Figure 4:**
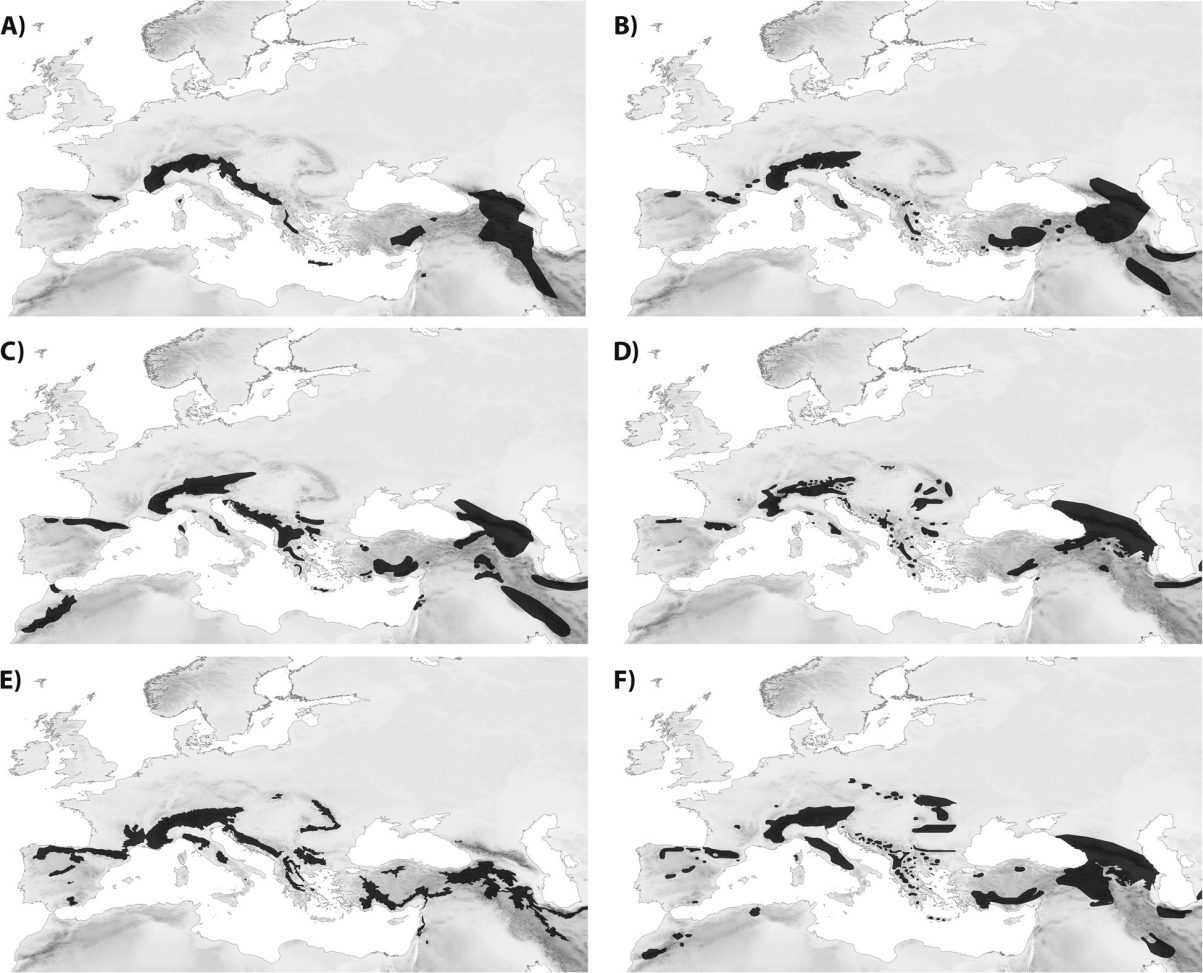
**Geographical distributions of six vertebrate species with palealpine distribution. (A)** Alpine long-eared bat *Plecotus macrobullaris*, **(B)** white-winged snowfinch *Montifringilla nivalis*, **(C)** alpine chough *Pyrrhocorax graculus*, **(D)** wallcreeper *Tichodroma muraria* (breeding areas), **(E)** snow vole *Chionomys nivalis* and **(F)** alpine accentor *Prunella collaris* (breeding areas). Map A was generated from data published by Alberdi et al. [14], and maps B-F were obtained from IUCN Red List of Threatened Species [69].

The distribution overlap analysis showed that *P. macrobullaris* shares its distribution pattern with five other vertebrates: the birds *Montifringilla nivalis*, *Pyrrhocorax graculus*, *Tichodroma muraria* and *Prunella collaris*, as well as the rodent *Chionomys nivalis*. Despite slight differences can be observed between the ranges of the mentioned species, they are all widely distributed but restricted to mountain environments (Figure 4). Therefore, the conclusions obtained from the ENM of *P. macrobullaris* are probably extensible to all of them. Additionally, as it is discussed below, all species exhibit similar ecological features. A handful of other birds, such as *Anthus spinoletta* (cSSI =0.58), *Pyrrhocorax pyrrhocorax* (0.57) or *Turdus torquatus* (0.53) were also linked to mountain environments, although their geographical distribution extends beyond the main mountain chains. The distribution range of the rest of analysed vertebrates showed lower resemblance with palealpine species (Figure 1).

The generated broad-scale model predicted that suitable areas for *P. macrobullaris* are found in almost all mountain ranges in the Western Palearctic, including some ranges where the species has not being hitherto reported. The species might be present in the Atlas Mts. in Morocco as well as in several mountain chains in the Iberian and Italian Peninsulas, as occurs with some other palealpine species (Figure 4). Two of the palealpine birds inhabit the Atlas while the Cantabrian Mts. and the Apennines host all the mentioned species. On the other hand, the presence of the species in the suitable mountain ranges of Scotland and Scandinavia seems implausible. Both ranges were covered by ice caps during Pleistocene glaciations [33], and the large unsuitable flatland separating them from the southern mountain ranges could have prevented their postglacial colonisation by palealpine species. Additionally, harder climatic conditions in winter than in Southern Europe might be another reason that hampers colonisation. This view is supported by the distribution of other palealpine species, which are absent from both Scotland and Scandinavia despite the relatively high mobility of most of them (Figure 4).

Our results differ from the previously published ecological niche model of *P. macrobullaris* in Switzerland [34], which predicted a distribution restricted to lower elevations than what our model did for the Alps. Nevertheless, both research are hardly comparable as they only used localities of colonies detected in buildings at valley bottoms for generating their ENM, which might led to a biased model of the distribution of *P. macrobullaris*, since the species also uses natural roosting resources [19], and it has been also reported above the treeline in the Alps [14].

### The role of topographic variables

**Figure 5:**
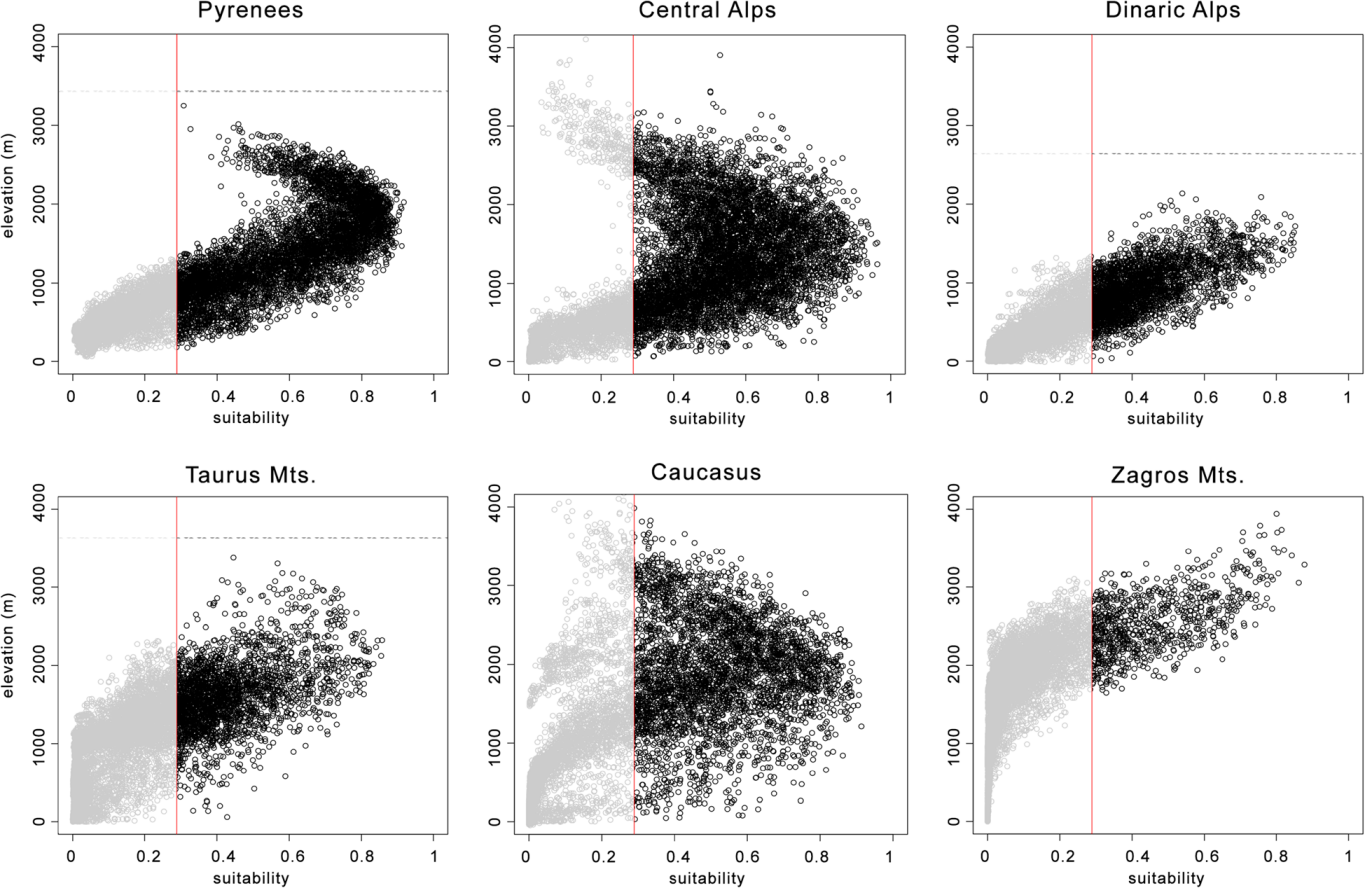
**Relationship between suitability and elevation in six mountain massifs.** Vertical red lines indicate the lower suitability boundary. Horizontal dotted lines indicate the maximum elevation of the mountain range.

Our models identified the abruptness of the landscape as the main constraint acting on the broad-scale distribution of *P. macrobullaris*, over-performing elevation in all cases. Unlike other variables linked to the elevation gradient (abruptness and slope), the effect of elevation itself was not consistent across spatial scales. Even though elevation is broadly used as a predictor in species distribution modelling [35], it does not directly affect the distribution of species on its own, but acts as a surrogate for several other habitat and climatic factors [36]. Thus, elevation may be a good predictor in regional-scale modelling [37], but its predictive ability decreases when moving from the regional to the continental scale, because it loses its ecological relevance (in the case of summer mean temperature, correlation value with elevation drops from ρ = −0.97 in the Pyrenees to ρ = −0.43 across the whole Palearctic). The great variation in the suitable elevation range between massifs (Figure 5) supports this statement.

Abruptness likely acts as a surrogate of several ecological variables. One of them is high rock availability, an important resource for palealpine species. In fact, the fine-scale modelling disclosed the importance of the proximity of rocky environments and the steepness of the terrain, surfacing the significance of rock walls and their debris. The five vertebrates identified as palealpine species depend on rocks for sheltering [38-40]. Cliffs and talus slopes are also a key roosting resource for *P. macrobullaris* [19], even though it also uses buildings for sheltering [34,41,42]. The relative area of rock outcrops is extensive at high mountain environments, favouring roost availability for saxicolous species. This behaviour probably allowed palealpine species to expand their ranges throughout the narrow and steep mountain massifs in the Western Palearctic. For instance, most snowfinches (genera *Montifringilla*, *Pyrgilauda* and *Onychostruthus*) roost on the ground and are behaviourally adapted to living in steppes [38], but only the saxicolous *M. nivalis* has expanded its distribution towards the European mountain massifs.

Another characteristic shared by all palealpine species is their preference for foraging in open areas, mainly in habitats with short grassy vegetation and rocks [18,43-45]. Climatic factors hamper the development of wood patches in alpine areas, providing suitable grounds for open-space foragers. Steep environments at lower elevation can also provide similar open habitats aided by the outcrop of bedrock and the decrease in soil thickness.

Finally, the high elevation range of mountain areas allows species to perform short-distance altitudinal migrations. Boreal species cannot avoid adverse atmospheric conditions by performing short migrations, and instead need to be physiologically adapted to extreme cold temperatures. In contrast, palealpine species tend to commute to lower elevations when conditions become harsher [39,46,47], and therefore physiological adaptations to the extreme cold may not be a compulsory requirement for them. Despite altitudinal migration have been documented in bats [48], such a behaviour has not been studied in *P. macrobullaris* so far. Nevertheless, it seems implausible bats could remain the whole year in alpine environments, and ecological features as well as comparative biogeography suggest that *P. macrobullaris* may actually perform seasonal altitudinal migrations [18].

### The role of climatic variables

Despite the lower contribution of climatic variables compared to topographic factors, the former also influenced the broad-scale model. Temperature mainly encompasses the altitudinal domains of the species’ distribution. Different climatic patterns along the elevation gradients of each mountain range (with differences of up to 13°C in mean summer temperature at the same elevation; Additional file 2: Figure S4) produced contrasting suitability-elevation relationships between mountain chains across the predicted range of *P. macrobullaris* (Figure 5). For instance, suitable areas in the Zagros Mountains (Iran) were restricted to areas above 1100 m, whereas near-sea-level areas of the Alps and the Dinaric Alps were considered suitable. This fact explains the poor predictive ability of elevation in the continental scale models. The fine-scale model also reflected the limiting effect of temperature in the Pyrenees, since no areas above 2600 m were considered suitable despite containing abundant steep areas.

Broad-scale modelling showed that *P. macrobullaris*, and by extension palealpine species, are able to cope with mean summer temperatures that span from 6 to 22°C (Figure 3B), revealing their eurithermic nature. Even though palealpine vertebrates were previously considered as cold-adapted species [21], their distribution is not restricted to cold environments, suggesting they are cold-tolerant rather than cold-adapted. Climatic models have predicted that alpine species like *P. graculus* and *M. nivalis* might extend their distribution to high latitude areas due to climate change [21]. Even though those areas might become climatically suitable for alpine species, our results suggest that such a scenario is highly improvable. We show that topography and its correlating ecological factors are shaping mostly the distribution of such species, and thus, the large unsuitable flatland between boreal areas and Southern European mountain ranges would hamper putative colonisation events. The wide thermal tolerance likely facilitated the colonisation of most of the mountain massifs in the Central and Southern Palearctic. Conversely, the southern isolated populations of an arctic-alpine vertebrate such as the rock ptarmigan (*Lagopus muta*) are limited to mountain ranges that hold extensive areas with summer mean temperatures below 10°C, i.e. the Pyrenees and the Alps in Europe [49].

### The human factor

In addition to topographic and climatic factors, human activities probably affected the distribution of palealpine vertebrates. Similar to other palealpine species [43,50,51], *P. macrobullaris* is also an open-space forager [18]. The natural treeline in the Pyrenees is expected to be around 2000–2200 m; however, historical landscape management for pasture has lowered the upper forest limit to around 1500 m [52]. It is noteworthy that the most suitable elevation range predicted by both models for the Pyrenees coincides with this modified belt (1500 – 2000 m), which suggests that it may offer the best ecological conditions for *P. macrobullaris* and other palealpine vertebrates in terms of greater food abundance and diversity [53] and milder climatic conditions than at higher elevations [54]. Hence, future changes in landscape management due to pastoral abandonment will likely affect the foraging ecology of *P. macrobullaris* [18] and other alpine vertebrates [44].

### Biotic interactions

The importance of biotic interactions in the distribution of species is a largely recognised phenomenon [55]. Processes like competitive exclusion [56] or predator–prey relationships [57] can affect the distribution of species, in addition to climatic and habitat-related factors. Accordingly, Rutishauser et al. [34] reported that the mostly allopatric distribution of *P. macrobullaris* and *P. austriacus* in Switzerland might be the result of competitive exclusion. In fact, both species share ecological traits [18,58,59]. However, under this scenario sympatric populations would be the exception rather than the rule and the niche of *P. macrobullaris* would expand where it is released from interspecific competition. Neither one nor the other seems to occur. There are broad areas where both species are found in sympatry, such as the Pyrenees (own data), the French Prealps [60] and the Dinaric Alps [61], and the ecological niche of *P. macrobullaris* as well as its link to mountain environments is kept beyond the range of *P. austriacus*, e.g. the eastern part of the distribution of *P. macrobullaris*. Competitive exclusion might occur in some specific areas like Switzerland, or across the altitudinal gradient in precise contact zones [62]. We do not discard that competitive exclusion shows up where resources are limiting, but in our opinion there are no signs indicating a general effect on the ecological niche and continental-level distribution of *P. macrobullaris*.

### The environmental niche of palealpine species

Our results show that climatic factors are not the main drivers of the distribution of palealpine vertebrates. Generally, the broad-scale geographic distribution of species tend to be limited by climatic variables [28], while non-climatic factors usually shape their distribution at the regional or local level [63]. However, our models show that in the case of palealpine species topographic factors act at a broader scale. Unlike arctic-alpine organisms, palealpine species are able to thrive in relatively warm as well as cold temperatures, and this eurithermicity is responsible for the limited ability of climatic variables to predict their distribution at the broad-scale.

Although palealpine species share the same biogeographic pattern driven by topographic factors, they are expected to show differential responses to climatic conditions. Variations in behaviour as well as geographical and altitudinal distribution suggest that some species cope better with cold than others, e.g. the white-winged snowfinch compared to the wallcreeper [23]. Differences in thermal tolerance between groups of organisms are also expected to occur due to differences in their ecology, reproduction and physiology. Major differences between birds and bats probably stem from their distinct circadian rhythm, thermoregulation abilities and reproductive systems. Birds are diurnal, homeothermic and oviparous animals [64], while bats are highly heterothermic nocturnal organisms with viviparous reproduction [65]. Even though facultative heterothermy may offer an advantage over homeothermic organisms when exploiting alpine environments, this advantage is only applicable to non-reproductive bats. Breeding females cannot decrease their body temperature as much as non-breeding bats due to the metabolic demands of foetal development and lactation [66], so they may be hindered from exploiting high mountain habitats. Consequently, elevational segregation might occur in the coldest regions of their distribution, which would explain the high number of *P. macrobullaris* maternity colonies found below the treeline in the Alps [41,42]. Nevertheless, such a pattern was not so noticeable in the slightly milder range of the Pyrenees [19]. Finally, models generated in this study reflect the environmental niche during the favourable season. During winter, palealpine vertebrates behave differently depending on species and taxa: bats hibernate while birds migrate to lower elevations.

## Conclusions

The use of multi-scale modelling allowed us to identify features shaping the biogeographic pattern shared by alpine vertebrates with wide geographic distribution, which could pave the way for future studies and help fine-tune conservation measures for this group of animals. The wide temperature tolerance identified in this study suggests that rather than physiological limitations relating to warmer conditions, ecological factors, such as changes in treeline elevation and the incursion of lowland species, may be the main challenges palealpine species will have to cope with under future conditions resulting from climate change [67]. Palealpine species are found in high-mountain environments not because they are constrained by cold temperatures, but because these cold-tolerant species find in alpine environments their preferred foraging and sheltering habitats, characterized by abundant open-space and rocky areas.

## Methods

### Distribution similarity

We calculated the distribution area overlap of *P. macrobullaris* and 503 European terrestrial vertebrates using the Sørensen Similarity Index (SSI - [68]) in order to identify the species sharing the distribution pattern with *P. macrobullaris*. We generated a reference GIS layer based on updated information about the distribution of *P. macrobullaris* [14] and compared it with breading season distribution layers of 203 mammals available at IUCN Red List [69] and 298 birds obtained from BirdLife International [70]. We also compared the distribution area of *P. macrobullaris* we generated with the area available at IUCN Red List to assess the uncertainty of the data and create a corrected SSI (cSSI) that reflects better the distribution similarities. Visual inspection of the geographical distributions of the species with the highest cSSI values showed that cSSI = 0.60 marks a resemblance threshold between widely distributed species limited to mountain ranges and species with a range that extends beyond the main mountain chains.

### Environmental Niche Modelling (ENM)

The extent of the broad-scale models was set as the Western Palearctic region (20–70° N, −20–60° E), while fine-scale models were limited to the Pyrenees and the surrounding area (40–42.1° N, −1.1–2.2 °E). All predictive models were generated with the presence-only species distribution modelling software Maxent (Maximum Entropy Algorithm) version 3.3.3 [71,72]. When running the software, we set maximum iterations to 5000, used 10000 background points, and ran 50 replicates of each model, following a resampling method randomly selecting a subsample of 25% of location records for model validation in each round. The output of all models was set in *raw* format in order to analyse them using the software ENMTools. Correlation between variables was analysed using ENMTools v1.3 [73], and the variable with *a priori* the highest ecological meaning was selected when the Spearman’s correlation coefficient (ρ) between two variables was greater than 0.65. The remaining spatial and statistical analyses were performed using ArcView GIS 3.2 and R 2.9.2 software (packages *raster* and *dismo*) available from CRAN [74].

### Broad-scale modelling

We used presence location records from all of the known distribution area of *P. macrobullaris* [14]. In order to minimise errors and ensure the best model performance, we took several measures following Merow et al. [75]. We applied 5 filters to all the available records with the following criteria: (1) *Molecular verification*: species identification was checked by molecular means to avoid misidentifications with morphologically similar congeners; (2) *Resolution*: maximum location uncertainty of 1 km; (3) *Pseudoreplication*: a minimum distance of 10 km between presence records was set based on the home-range information of the species [60,76]; (4) *Record source-type homogeneity*: similar amount of different record types (e.g. roost observations, net captures); and (5) *Geographical homogeneity*: similar amount of records in each geographic area. Sampling effort East of the Bosphorus has been considerably lower than in Europe, and the number of European records were four-fold the number of eastern locations after applying the filtering. In order to correct the sampling bias [77], a bias grid was introduced to the algorithm where the relative sampling probability West of the Bosphorus was considered four times higher than the eastern area. This helped outweigh the relative value of Anatolia, the Near East and the Caucasus compared to Europe. Spatial autocorrelation analyses were discarded because the disjunct distribution of alpine species is actually grouped in clusters limited to high mountain massifs.

We modelled the distribution of *P. macrobullaris* at a resolution of 30-arc-second (approximately 1 km) using 74 presence records. In order to avoid large unsuitable areas, such as the Siberian steppe or Sahara desert, that could yield artificially inflated model evaluations [78,79], we set a 500 km buffer around presence locations in the calibration area (Figure 2); i.e. the area from which background points used for generating the model were randomly selected. After filtering highly correlated predictors, we used three types of variables (6 climatic, 2 topographic and 1 habitat-related – Table 3) for generating 90 different models (each one including 50 replicates) using different variable combinations and regularization parameters. Climatic variables and elevation data were obtained from WorldClim database [80]. Land Cover data was obtained from GLCNMO [81]. Abruptness of landscape (ABR) was calculated from the elevation grid computing the maximum elevation difference within a 5 km buffer from each cell. Land cover categories were reclassified to 6 categories: forests, open natural areas, agricultural areas, urban areas, ice/snow and water.

**Table 3:** **Characteristics of the variables used in the two modelling scales**

**Broad-scale modelling**				
**Predictor**	**Predictor description**	**Class**	**Resolution**	**Source**
**ABR**	Abruptness	Topographic	30 arc-sec	Generated from elevation data
**ELEV**	Elevation	Topographic	30 arc-sec	Wordclim SRTM
**B4**	Temperature seasonality	Climatic	30 arc-sec	Worldclim Bioclim 4
**B8**	Mean temperature of the wettest quarter	Climatic	30 arc-sec	Worldclim Bioclim 8
**B10**	Mean temperature of the warmest quarter	Climatic	30 arc-sec	Worldclim Bioclim 10
**B12**	Annual precipitation	Climatic	30 arc-sec	Worldclim Bioclim 12
**B15**	Precipitation seasonality	Climatic	30 arc-sec	Worldclim Bioclim 15
**B17**	Precipitation of the Driest Quarter	Climatic	30 arc-sec	Worldclim Bioclim 17
**LAND**	Land cover	Habitat	30 arc-sec	GLCNMO
**Fine-scale modelling**				
**Predictor**	**Description**	**Class**	**Resolution**	**Source**
**ELEV**	Elevation	Topographic	100 m	SRTM 90 m DEM (CGIAR-CSI)
**SLO**	Slope	Topographic	100 m	Generated from elevation data
**ORI**	Orientation	Topographic	100 m	Generated from elevation data
**DIS-ROCK**	Distance to rock areas	Habitat	100 m	Obtained from Corine LandCover 2006
**DIS-FOREST**	Distance to forest areas	Habitat	100 m	Obtained from Corine LandCover 2006
**DIS-URBAN**	Distance to urban areas	Habitat	100 m	Obtained from Corine LandCover 2006

### Fine-scale modelling

We used roost location data for modelling the fine-scale environmental niche of *P. macrobullaris* (Additional file 2: Figure S1). Roosts were identified using the *homing-in* radio-tracking method [19]. Only roosts located in natural structures were used, as they represent the original as well as the predominant roost type of *P. macrobullaris* [19]. In order to minimise pseudoreplication, one roost for each grid cell was used for the analysis, resulting in a total of 43 records. We used 6 variables (3 topographic and 3 habitat-related – Table 3) to generate 45 different models. Climatic variables were not used in the fine-scale modelling for being highly correlated with elevation. All variables were edited and homogenised to 100 m resolution. Elevation data was obtained from CGIAR-CSI [82], and the rest of topographic variables were derived from it. Habitat variables were obtained from Corine Land Cover 2006 [83] and categories were reclassified in ArcView GIS 3.2.

Abruptness was used in the broad-scale modelling for reflecting the elevation range available for the bats in each geographic location, and it was based on the home-range information of *P. macrobullaris* [60,76]. Conversely, we used slope in the fine-scale modelling because we considered it might play a key role providing suitable rock roosts to *P. macrobullaris* [19].

### Model evaluation and selection

Models were evaluated using both threshold-independent and dependent means that deal with several aspects of model performance, including model accuracy, model complexity and prediction success [84-86]. Accuracy was evaluated using the Area Under the Curve (AUC) of the Receiver Operator Characteristics (ROC) [32,87-90]. Corrected Akaike’s Information Criteria (AICc) [91] was used for evaluating model complexity in ENMTools. For that task a single model out of the 50 replicates was selected using a Principal Components Analysis (PCA), in order to identify the replicate with the closest values to the average. The PCA was performed using five matrices: regularised training gain, test gain, test AUC, 10 percentile training presence logistic threshold and 10 percentile training presence area. We also used the threshold-dependent minimal predicted area (MPA) evaluator [92]. Finally, we validated the best broad-scale model using all published distribution records (349) of *P. macrobullaris* [14].

### Model output and variable importance

The best models were repeated and plotted in a logistic format to provide the estimates of the probability of occurrence as predicted by the variables in a map [72,93]. We used the threshold selection method max SSS to discern suitable and unsuitable areas [94]. The relative importance of each variable was checked using heuristic (percent contribution), permutation (permutation importance) and jackknife approaches (Table 2) [95,96]. Response curves for the best explanatory variables were plotted in order to determine the response of suitability values to changes in specific predictors, and thus identify the most suitable conditions for the species (Figure 3).

## Additional files

Additional file 1: Two tables showing the composition, evaluation and variable contribution ranks of the 90 broad-scale models **(Table S1)** and 45 fine-scale models **(Table S2)**. Additional file 2:
**Figure S1.** map showing the roosting records used for the fine scale-modelling, **Figure S2.** results of the jackknife tests of variable importance of the best broad-scale model, **Figure S3.** results of the jackknife tests of variable importance of the best fine-scale model, and **Figure S4.** mean summer temperatures at different elevations and mountain ranges.
