# Supplementary material for: Unveiling the factors shaping the distribution of widely distributed alpine vertebrates, using multi-scale ecological niche modelling of the bat Plecotus macrobullaris
Source: Front Zool. 2014 Oct 29;11:77. doi: 10.1186/s12983-014-0077-6 (PMC4226887; doi:10.1186/s12983-014-0077-6)
Supplement: Additional file 1: — Two tables showing the composition, evaluation and variable contribution ranks of the 90 broad-scale models (Table S1) and 45 fine-scale models (Table S2). [file 12983_2014_77_MOESM1_ESM.doc]

**Additional file 1**

**Table S1**. Broad-scale models. For each model are shown the number of variables, the name of the variables, the regularization value (β) and three evaluation metrics: the area under the curve (AUC), the minimal predicted area (MPA) and the corrected Akaike Information Criteria (AICc)

| **Model** | **# V** | **Variables** | **β** | **AUC** | **MPA** | **AICc** |
| --- | --- | --- | --- | --- | --- | --- |
| **Climatic** | | | | | | |
| **1** | 6 | B4, B8, B10, B10, B15, B17 | 1 | 0.8716 | 0.318 | 2585.8857 |
| **2** |  |  | 2 | 0.8646 | 0.3628 | 2661.1093 |
| **3** |  |  | 4 | 0.8581 | 0.3809 | 2646.0533 |
| **4** | 4 | B8, B10, B12, B17 | 1 | 0.8812 | 0.3383 | 2688.1285 |
| **5** |  |  | 2 | 0.8668 | 0.3486 | 2667.6994 |
| **6** |  |  | 4 | 0.8613 | 0.3902 | 2622.2708 |
| **7** | 5 | B8, B10, B12, B15, B17 | 1 | 0.8789 | 0.3332 | 2690.8984 |
| **8** |  |  | 2 | 0.8708 | 0.3542 | 2661.0886 |
| **9** |  |  | 4 | 0.8649 | 0.3641 | 2664.3820 |
| **10** | 5 | B4, B10, B12, B15, B17 | 1 | 0.8802 | 0.2972 | 2619.7250 |
| **11** |  |  | 2 | 0.8766 | 0.3124 | 2714.7772 |
| **12** |  |  | 4 | 0.8645 | 0.3543 | 2717.7897 |
| **13** | 4 | B10, B12, B15, B17 | 1 | 0.8745 | 0.2977 | 2756.6882 |
| **14** |  |  | 2 | 0.8813 | 0.323 | 2782.5089 |
| **15** |  |  | 4 | 0.8629 | 0.3666 | 2744.2974 |
| **16** | 4 | B4, B10, B12, B17 | 1 | 0.8817 | 0.3469 | 2722.7548 |
| **17** |  |  | 2 | 0.8712 | 0.3454 | 2629.4739 |
| **18** |  |  | 4 | 0.8571 | 0.3786 | 2599.8300 |
| **Topographic** | | | | | | |
| **19** | 2 | ELEV, ABR | 1 | 0.8732 | 0.3657 | 2434.7099 |
| **20** |  |  | 2 | 0.8662 | 0.343 | 2478.7364 |
| **21** |  |  | 4 | 0.8654 | 0.3796 | 2527.68 |
| **Climatic + Topographic** | | | | | | |
| **22** | 8 | B4, B8, B10, B12, B15, B17, ELEV, ABR | 1 | 0.905 | 0.2005 | 2542.8793 |
| **23** |  |  | 2 | 0.9024 | 0.2523 | 2481.2998 |
| **24** |  |  | 4 | 0.896 | 0.2718 | 2441.1811 |
| **25** | 7 | B4, B8, B10, B12, B15, B17, ABR | 1 | 0.9027 | 0.2122 | 2422.9398 |
| **26** |  |  | 2 | 0.9112 | 0.2376 | 2409.3266 |
| **27** |  |  | 4 | 0.8977 | 0.2551 | 2410.0908 |
| **28** | 7 | B4, B8, B10, B12, B15, B17, ELEV | 1 | 0.8944 | 0.2539 | 2794.8147 |
| **29** |  |  | 2 | 0.8821 | 0.2953 | 2692.5058 |
| **30** |  |  | 4 | 0.8861 | 0.3185 | 2555.0349 |
| **31** | 5 | B10, B12, B15, B17, ELEV | 1 | 0.8687 | 0.2909 | 2622.6515 |
| **32** |  |  | 2 | 0.8865 | 0.3081 | 2564.912 |
| **33** |  |  | 4 | 0.869 | 0.3202 | 2568.8443 |
| **34** | 5 | B10, B12, B15, B17, ABR | 1 | 0.9046 | 0.2452 | 2591.3639 |
| **35** |  |  | 2 | 0.8943 | 0.2738 | 2505.6425 |
| **36** |  |  | 4 | 0.8923 | 0.2815 | 2472.5665 |
| **37** | 6 | B8, B10, B12, B15, B17, ABR | 1 | 0.8914 | 0.2404 | 2471.6527 |
| **38** |  |  | 2 | 0.8999 | 0.2424 | 2465.4122 |
| **39** |  |  | 4 | 0.9001 | 0.2765 | 2463.3529 |
| **40** | 6 | B4, B10, B12, B15, B17, ABR | 1 | 0.9052 | 0.2557 | 2480.8631 |
| **41** |  |  | 2 | 0.898 | 0.2471 | 2449.4132 |
| **42** |  |  | 4 | 0.8952 | 0.2877 | 2476.4189 |
| **43** | 5 | B4, B8, B10, B17, ABR | 1 | 0.9017 | 0.2318 | 2492.464 |
| **44** |  |  | 2 | 0.895 | 0.2431 | 2404.6237 |
| **45** |  |  | 4 | 0.8967 | 0.3572 | 2418.6237 |
| **Climatic + Habitat** | | | | | | |
| **46** | 7 | B4, B8, B10, B12, B15, B17, LAND | 1 | 0.8993 | 0.2713 | 2426.4305 |
| **47** |  |  | 2 | 0.8821 | 0.318 | 2486.7522 |
| **48** |  |  | 4 | 0.8535 | 0.3345 | 2498.7075 |
| **49** | 6 | B8, B10, B12, B15, B17, LAND | 1 | 0.8792 | 0.297 | 2509.8652 |
| **50** |  |  | 2 | 0.8752 | 0.3209 | 2475.5632 |
| **51** |  |  | 4 | 0.8769 | 0.3119 | 2480.3945 |
| **52** | 5 | B4, B10, B12, B15, B17, LAND | 1 | 0.8892 | 0.2378 | 2472.1983 |
| **53** |  |  | 2 | 0.8494 | 0.2593 | 2461.8294 |
| **54** |  |  | 4 | 0.8745 | 0.286 | 2487.1674 |
| **55** | 5 | B10, B12, B15, B17, LAND | 1 | 0.8802 | 0.267 | 2517.9123 |
| **56** |  |  | 2 | 0.876 | 0.2782 | 2503.8898 |
| **57** |  |  | 4 | 0.8764 | 0.3029 | 2500.1469 |
| **58** | 4 | B10, B12, B17, LAND | 1 | 0.8826 | 0.2382 | 2499.8646 |
| **59** |  |  | 2 | 0.8878 | 0.2599 | 2490.6786 |
| **60** |  |  | 4 | 0.8776 | 0.315 | 2493.6051 |
| **Topographic + Habitat** | | | | | | |
| **61** | 3 | ELEV, ABR, LAND | 1 | 0.8734 | 0.3209 | 2333.0673 |
| **62** |  |  | 2 | 0.8604 | 0.3288 | 2373.3808 |
| **63** |  |  | 4 | 0.8711 | 0.3188 | 2400.0137 |
| **64** | 2 | ELEV, LAND | 1 | 0.794 | 0.4625 | 2466.8868 |
| **65** |  |  | 2 | 0.7926 | 0.4694 | 2496.4921 |
| **66** |  |  | 4 | 0.7881 | 0.4643 | 2542.1828 |
| **67** | 2 | ABR, LAND | 1 | 0.8668 | 0.3131 | 2373.2762 |
| **68** |  |  | 2 | 0.8764 | 0.3205 | 2379.0112 |
| **69** |  |  | 4 | 0.8713 | 0.3163 | 2394.0448 |
| **Climatic + Topographic + Habitat** | | | | | | |
| **70** | 9 | B4, B8, B10, B12, B15, B17, ELEV, ABR, LAND | 1 | 0.9004 | 0.1776 | 2494.2734 |
| **71** |  |  | 2 | 0.8994 | 0.2242 | 2369.9424 |
| **72** |  |  | 4 | 0.8987 | 0.2447 | 2320.4055 |
| **73** | 8 | B4, B8, B10, B12, B15, B17, ABR, LAND | 1 | 0.903 | 0.1785 | 2334.3659 |
| **74** |  |  | 2 | 0.8938 | 0.2228 | 2344.8563 |
| **75** |  |  | 4 | 0.9035 | 0.254 | 2323.0707 |
| **76** | 8 | B4, B8, B10, B12, B15, B17, ELEV, LAND | 1 | 0.8974 | 0.223 | 2473.7872 |
| **77** |  |  | 2 | 0.8841 | 0.2749 | 2423.2009 |
| **78** |  |  | 4 | 0.8561 | 0.319 | 2388.5569 |
| **79** | 6 | B10, B12, B15, B17, ABR, LAND | 1 | 0.8726 | 0.2792 | 2392.6485 |
| **80** |  |  | 2 | 0.8762 | 0.2822 | 2361.1236 |
| **81** |  |  | 4 | 0.8746 | 0.2935 | 2418.8629 |
| **82** | 6 | B10, B12, B15, B17, ELEV, LAND | 1 | 0.8609 | 0.3153 | 2405.6119 |
| **83** |  |  | 2 | 0.8831 | 0.2948 | 2407.9747 |
| **84** |  |  | 4 | 0.8753 | 0.2989 | 2388.3084 |
| **85** | 7 | B4, B10, B12, B15, B17, ABR, LAND | 1 | 0.912 | 0.2086 | 2330.3513 |
| **86** |  |  | 2 | 0.9053 | 0.2107 | 2309.4962 |
| **87** |  |  | 4 | 0.8991 | 0.2777 | 2323.5367 |
| **88** | 7 | B4, B10, B12, B15, B17, ELEV, LAND | 1 | 0.8749 | 0.2567 | 2378.5873 |
| **89** |  |  | 2 | 0.8855 | 0.2756 | 2386.9786 |
| **90** |  |  | 4 | 0.8723 | 0.2825 | 2389.5594 |

**Table S2**. Fine-scale models. For each model are shown the number of variables, the name of the variables, the regularization value (β) and three evaluation metrics: the area under the curve (AUC), the minimal predicted area (MPA) and the corrected Akaike Information Criteria (AICc)

| **Model** | | | **# V** | **Variables** | **β** | **AUC** | **MPA** | **AICc** |
| --- | --- | --- | --- | --- | --- | --- | --- | --- |
| **Topographic** | | | | | | | | |
| **1** | 3 | elev, slop, ori | | | 1 | 0.9294 | 0.1381 | 1087.2041 |
| **2** |  |  |  |  | 2 | 0.922 | 0.1755 | 1088.3409 |
| **3** |  |  |  |  | 4 | 0.912 | 0.2079 | 1091.0634 |
| **4** | 2 | elev, slop | | | 1 | 0.9298 | 0.1574 | 1108.6065 |
| **5** |  |  |  |  | 2 | 0.9243 | 0.1657 | 1086.4559 |
| **6** |  |  |  |  | 4 | 0.9208 | 0.2067 | 1093.5073 |
| **7** | 2 | elev, ori | | | 1 | 0.8748 | 0.2359 | 1122.7974 |
| **8** |  |  |  |  | 2 | 0.8645 | 0.2487 | 1133.3629 |
| **9** |  |  |  |  | 4 | 0.846 | 0.2537 | 1137.1957 |
| **10** | 2 | slop, ori | | | 1 | 0.8635 | 0.3301 | 1113.8787 |
| **11** |  |  |  |  | 2 | 0.8751 | 0.3509 | 1116.7807 |
| **12** |  |  |  |  | 4 | 0.8556 | 0.331 | 1059.8584 |
| **Habitat** | | | | | | | | |
| **13** | 3 | dis-for, dis-urban, dis-rock | | | 1 | 0.8968 | 0.2449 | 1161.1387 |
| **14** |  |  |  |  | 2 | 0.8718 | 0.2492 | 1146.7337 |
| **15** |  |  |  |  | 4 | 0.8732 | 0.2723 | 1128.3550 |
| **16** | 2 | dis-for, dis-urban | | | 1 | 0.8332 | 0.3841 | 1210.2592 |
| **17** |  |  |  |  | 2 | 0.8187 | 0.4331 | 1161.3130 |
| **18** |  |  |  |  | 4 | 0.7926 | 0.4527 | 1169.7824 |
| **19** | 2 | dis-urban, dis-rock | | | 1 | 0.8943 | 0.2471 | 1321.0146 |
| **20** |  |  |  |  | 2 | 0.8797 | 0.2495 | 1148.9252 |
| **21** |  |  |  |  | 4 | 0.8796 | 0.2695 | 1136.1749 |
| **22** | 2 | dis-for, dis-rock | | | 1 | 0.8596 | 0.2921 | 1165.6367 |
| **23** |  |  |  |  | 2 | 0.8617 | 0.2871 | 1145.8394 |
| **24** |  |  |  |  | 4 | 0.8556 | 0.3076 | 1152.4365 |
| **Topographic + Habitat** | | | | | | | | |
| **25** | 6 | elev, slop, ori, dis-for, dis-urban, dis-rock | | | 1 | 0.9429 | 0.0871 | 1289.9253 |
| **26** |  |  |  |  | 2 | 0.9418 | 0.1306 | 1059.8584 |
| **27** |  |  |  |  | 4 | 0.9176 | 0.1604 | 1082.9395 |
| **28** | 5 | elev, slop, dis-for, dis-urban, dis-rock | | | 1 | 0.9426 | 0.0933 | 1290.2809 |
| **29** |  |  |  |  | 2 | 0.9399 | 0.1392 | 1068.5225 |
| **30** |  |  |  |  | 4 | 0.9249 | 0.1679 | 1079.6535 |
| **31** | 4 | elev, slop, dis-urban, dis-rock | | | 1 | 0.9560 | 0.1026 | 1199.8610 |
| **32** |  |  |  |  | 2 | 0.9046 | 0.1565 | 1064.7293 |
| **33** |  |  |  |  | 4 | 0.9144 | 0.1807 | 1081.6386 |
| **34** | 4 | elev, slop, dis-for, dis-rock | | | 1 | 0.9257 | 0.1168 | 1076.8610 |
| **35** |  |  |  |  | 2 | 0.9361 | 0.1372 | 1070.0998 |
| **36** |  |  |  |  | 4 | 0.921 | 0.1735 | 1092.3558 |
| **37** | 3 | elev, slop, dis-rock | | | 1 | 0.9303 | 0.1411 | 1102.8659 |
| **38** |  |  |  |  | 2 | 0.9292 | 0.1501 | 1076.1372 |
| **39** |  |  |  |  | 4 | 0.9202 | 0.1872 | 1091.2960 |
| **40** | 3 | elev, slop, dis-urban | | | 1 | 0.9381 | 0.1219 | 1075.8442 |
| **41** |  |  |  |  | 2 | 0.9309 | 0.1462 | 1082.7430 |
| **42** |  |  |  |  | 4 | 0.9166 | 0.1997 | 1097.2855 |
| **43** | 3 | elev, slop, dis-for | | | 1 | 0.9339 | 0.1476 | 1100.0101 |
| **44** |  |  |  |  | 2 | 0.9431 | 0.1656 | 1079.4184 |
| **45** |  |  |  |  | 4 | 0.9245 | 0.1848 | 1093.6155 |
