# Supplementary material for: Unveiling the factors shaping the distribution of widely distributed alpine vertebrates, using multi-scale ecological niche modelling of the bat Plecotus macrobullaris
Source: Front Zool. 2014 Oct 29;11:77. doi: 10.1186/s12983-014-0077-6 (PMC4226887; doi:10.1186/s12983-014-0077-6)

**Additional file 2**

**Figure S1**. Roosting records obtained by Alberdi et al. (2014) used in the fine-scale modelling.


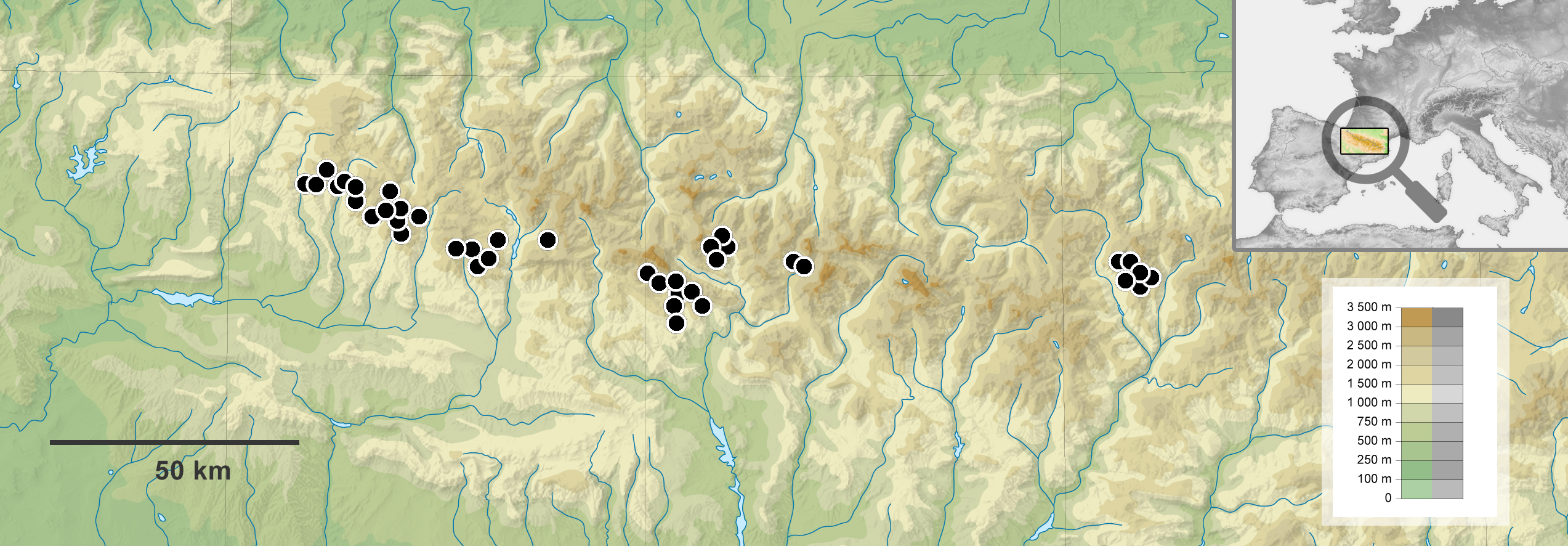


**Figure S2**. Results of the jackknife tests of variable importance of the best broad-scale model.

**
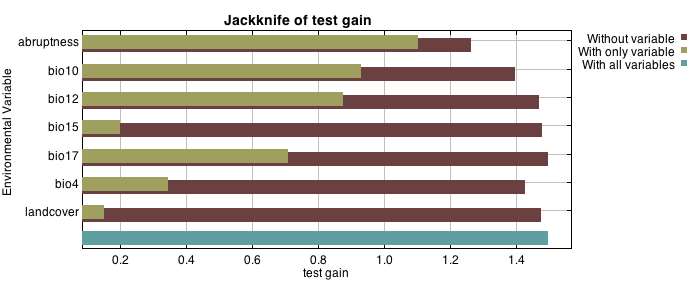

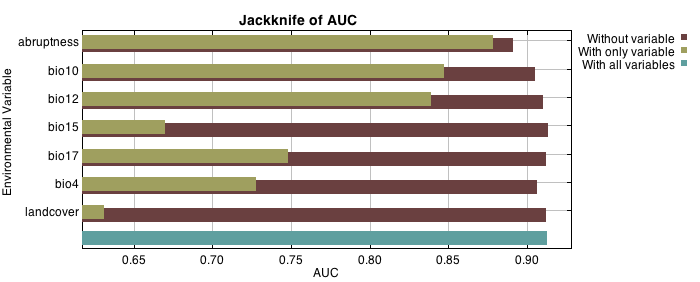

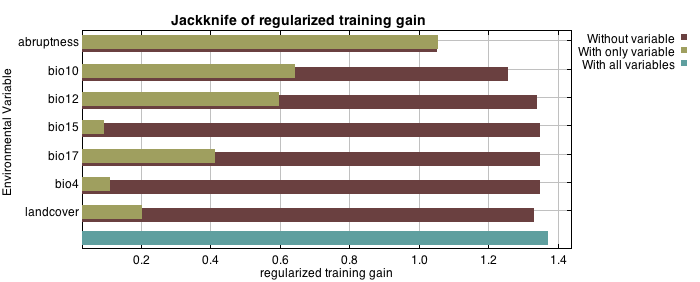
**

**Figure S3**. Results of the jackknife tests of variable importance of the best fine-scale model.


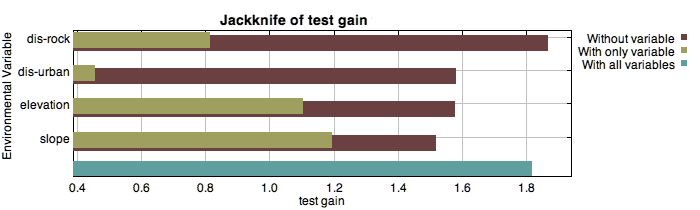

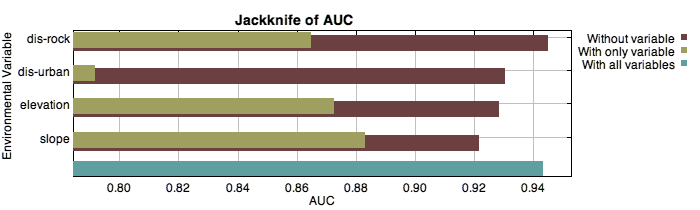

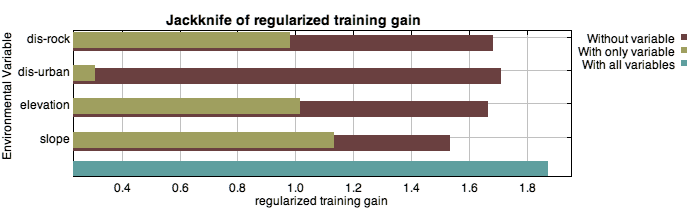


**Figure S4**. Mean summer temperatures at different elevations and mountain ranges. Data obtained from the variable BIO10 (mean temperature of the warmest quartet) of the climatic envelope Bioclim (<http://www.worldclim.org/bioclim>) by randomly selecting 500 points at different elevations (500-2500 m asml) for each mountain range.


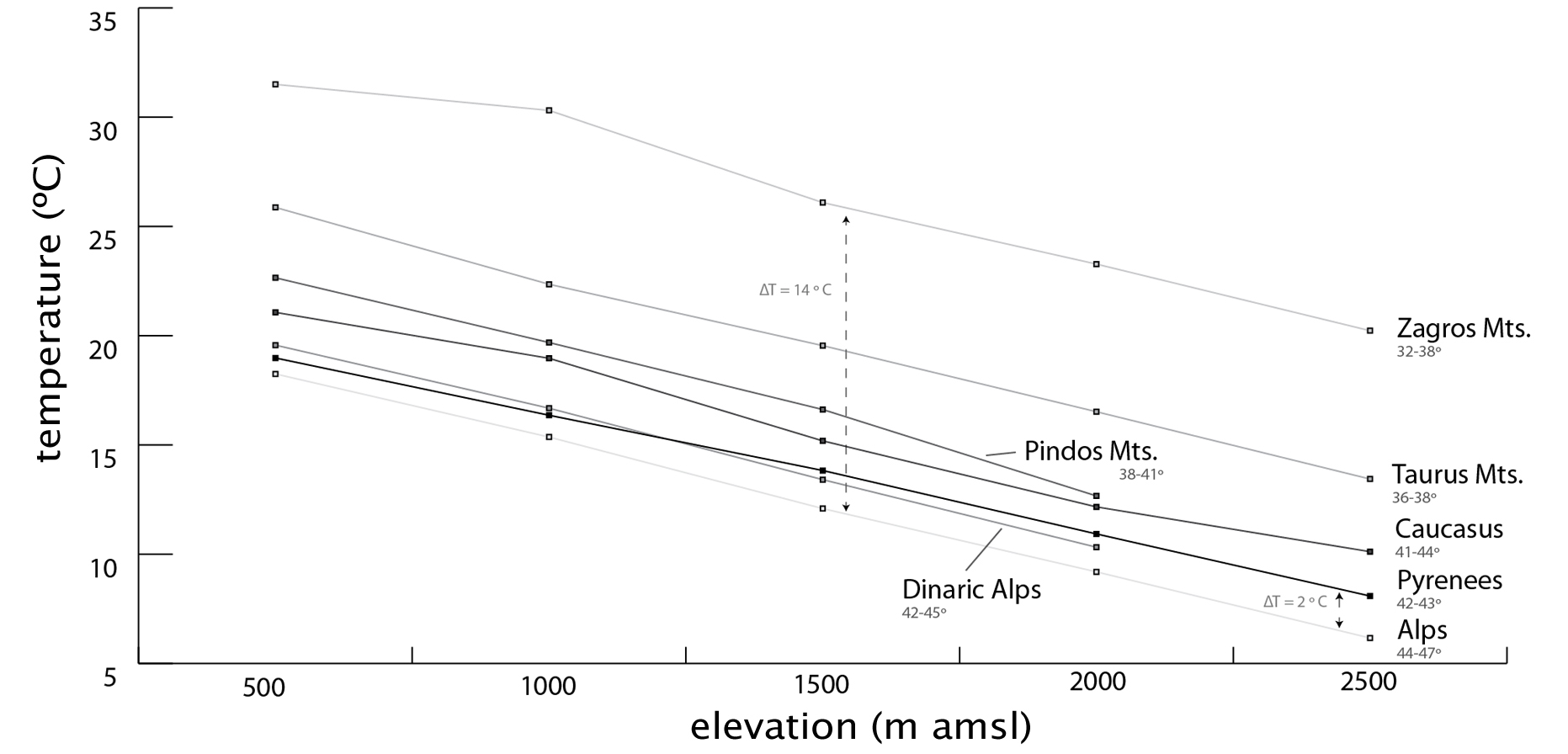

Supplement: Additional file 2: — Figure S1. map showing the roosting records used for the fine scale-modelling, Figure S2. results of the jackknife tests of variable importance of the best broad-scale model, Figure S3. results of the jackknife tests of variable importance of the best fine-scale model, and Figure S4. mean summer temperatures at different elevations and mountain ranges. [file 12983_2014_77_MOESM2_ESM.doc]
